# Mainstreaming control interventions for neglected tropical diseases into the health system: a scoping review protocol

**DOI:** 10.1136/bmjopen-2024-090252

**Published:** 2024-12-27

**Authors:** Awraris Hailu Bilchut, Teshome Gebre, Fikre Seife, Wendemagegn Enbiale, Adugna Tamiru, Tsegaye Yohanes, Amsayaw Yohannes, Yosef Adane, Behailu Merdekios, Biruck Kebede, Fikreab Kebede, Kebede Deribe, Jennifer Evans, Matthew J Burton, Esmael Habtamu

**Affiliations:** 1 School of Public Health, Asrat Woldeyes Health Sciences College, Debre Berhan University, Debre Berhan, Ethiopia; 2 Eyu-Ethiopia: Eye Health Research, Training & Service Centre, Bahir Dar, Ethiopia; 3 The Task Force for Global Health, Addis Ababa, Ethiopia; 4 National NTD Program, Disease Prevention and Control Directorate, Ministry of Health - Ethiopia, Addis Ababa, Ethiopia; 5 College of Medicine and Health Sciences, Bahir Dar University, Bahir Dar, Ethiopia; 6 Collaborative Research and Training Center for NTDs (CRTC-NTDs), Arba Minch University, Arba Minch, Ethiopia; 7 Research and Community Services, Arba Minch University, Arba Minch, Ethiopia; 8 RTI International, Addis Ababa, Ethiopia; 9 Children’s Investment Fund Foundation, Addis Ababa, Ethiopia; 10 Clinical Research Department, Faculty of Infectious and Tropical Diseases, London School of Hygiene & Tropical Medicine, London, UK; 11 National Institute for Health Research Biomedical Research Centre for Ophthalmology, Moorfields Eye Hospital NHS Foundation Trust and UCL Institute of Ophthalmology, London, UK

**Keywords:** Neglected Diseases, HEALTH SERVICES ADMINISTRATION & MANAGEMENT, Mass Drug Administration, PUBLIC HEALTH

## Abstract

**Abstract:**

**Introduction:**

The WHO neglected tropical diseases (NTD) roadmap (2021–2030) proposed a shift in approach to addressing NTDs through accountability for impact, implementing integration across NTDs, mainstreaming in national health systems and ensuring country ownership. However, a major challenge has been the dearth of evidence on how to implement this shift in a resource-limited setting. The objective of this scoping review is to understand the extent and type of evidence on the mainstreaming or integration of programmes and/or interventions against NTDs into the national health system.

**Methods:**

Research articles of any methodology, series, evaluations and reports published from 1995 in the English language on any peer-reviewed platforms, and WHO, universities and government or implementing partners websites that report and discuss mainstreaming or integration implementation practices or experiences of any interventions against NTDs into the health system will be included. The main databases searched on 30 October 2024 were Medical Literature Analysis and Retrieval System Online (Ovid), Embase (Ovid), Global Health (Ovid), Cochrane Database of Systematic Reviews and CENTRAL on the Cochrane Library (Wiley), and WHO Global Index Medicus. Following piloting, screening and data extraction will be undertaken independently by at least two people. Any disagreements that arise between the reviewers at each stage of the screening and selection process will be resolved through discussion, or with an additional reviewer/s. Thematic analysis will be conducted guided by the project INTEGRATE framework and the Primary Health Care Performance Initiative conceptual framework. Key information or evidence relevant to the mainstreaming of interventions against NTDs into the health system such as mainstreamed interventions and the level of health system where the mainstreaming occurred, the effectiveness of the mainstreaming, contextual differences in health system organisation between settings, and challenges and promising practices will be summarised.

**Ethics and dissemination:**

Ethical approval will not be sought as our review will only include published data. The findings will be published in a peer-reviewed journal and will be disseminated at national and international research and programme review platforms.

**STRENGTHS AND LIMITATIONS OF THIS STUDY**

- The search will be guided and implemented by two experienced evidence synthesis and information specialists.
- The reviewers will bring diverse perspectives from various population backgrounds and academic disciplines, which helps mitigate potential biases in interpreting the studies under review.
- The scoping review will only include those that are published in the English language, introducing an element of inclusion bias.
- We will only focus on publications and reports on peer-reviewed platforms and websites of WHO, universities, government and implementing partners, excluding information produced outside of the traditional publishing and distribution channels, which may lead to publication bias.
- A potential limitation could be a small number of publications identified that report mainstreaming of NTD programme or interventions.

## Introduction

 Neglected tropical diseases (NTDs) are a diverse group of diseases that are common in tropical and subtropical regions, primarily affecting people living in marginalised situations with limited access to sanitation and health facilities.^1 2^ These diseases are characterised by considerable disability with 14.5 million disability-adjusted life years (DALYs) associated with a significant reduction in both physical and psychosocial functioning.^3^ Significant progress has been made in the last decade in the prevention, control, elimination and eradication of NTDs globally. As of 2023, a total of 50 countries worldwide have eliminated at least one NTD.^4^ Between 2010 and 2022, the number of people requiring interventions against NTDs decreased by 26%.^4^ However, this reduction does not provide the required trajectory to achieve the WHO NTD roadmap (2021–2030) target of a 90% reduction by 2030.^1^ NTDs persist as significant health challenges affecting about 1.62 billion people worldwide.^4^ These diseases reflect inequitable access to quality healthcare services and impend progress towards achieving universal health coverage (UHC) targets.

The WHO NTD roadmap proposed a fundamental shift in the approach to tackling NTDs by increasing accountability, intensifying cross-cutting approaches through implementing integrated approaches, mainstreaming activities within national health systems, coordinating efforts across sectors and increasing country ownership.^1^ Integrated and mainstreamed approaches improve sustainability, efficiency and patients’ equitable access to all aspects of treatment, care and support.^1^ In alignment with the proposed shift, NTD-affected countries, such as Ethiopia, are actively working to mainstream NTD control activities within the national health system. However, a major challenge has been the dearth of evidence on how to implement this shift in a resource-limited setting.

The objective of this scoping review is to assess the extent and type of evidence on the mainstreaming or integration of programmes and/or interventions against NTDs into the health system. Mainstreaming in this review is defined, as per the description in the WHO NTD roadmap, as ‘the planning and delivery of interventions against NTDs through the national health system infrastructure to build capacity and contribute to sustainable, efficient disease prevention and control’.^1^ Literature that fits into this definition despite not using the term ‘mainstreaming’ or ‘integration’ (instead using other terms such as assimilation, combination, transitioning) will be included in this scoping review. A preliminary search of relevant databases (Medical Literature Analysis and Retrieval System Online (MEDLINE), Cochrane Library) and registers (PROSPERO) did not identify any current or ongoing scoping or systematic reviews on this topic. Our group has also registered a systematic review on this topic (PROSPERO, registration number: CRD42022362624), which will be conducted after the current scoping review, depending on the nature and extent of the literature.

The potential evidence to be generated from this scoping review will support NTD endemic countries’ efforts to achieve the envisaged targets of the WHO NTD roadmap. It will identify promising practices, challenges and success from different settings allowing endemic countries to learn from each other on how to intensify cross-cutting actions including the integration, mainstreaming and coordination of interventions, programmes and stakeholders against NTDs.^1^ It will also identify potential evidence and specific questions for subsequent systematic reviews, as well as evidence gaps.

### Objectives and review questions

The objective of this scoping review is to assess the extent and type of evidence on the mainstreaming or integration of programmes and/or interventions against NTDs into the health system. The review questions are:

- What is the nature and extent of the evidence on mainstreaming or integration efforts of programmes and/or interventions against NTDs into the health system across endemic settings globally?
- What key provider and user related challenges and promising practices limit and facilitate mainstreaming or integration of programmes and/or interventions against NTDs across different NTD endemic settings?

## Methods and analysis

### Protocol and registration

We will conduct this scoping review in accordance with the Joanna Briggs Institute (JBI) methodology for scoping reviews,^5^ and report in line with the Preferred Reporting Items for Systematic Reviews and Meta-Analyses extension for Scoping Reviews (PRISMA-ScR).^6^ This review is appropriate to be conducted as a scoping review, as it aims to explore the extent and type of evidence available on mainstreaming or integration of programme or interventions against NTDs into the health system and identify challenges and promising practices to inform and support future implementations and thereby support the global effort to control, eliminate and eradicate NTDs by 2030. The protocol was registered on Open Science Framework on 31 October 2024 (https://osf.io/g35n8). The whole study process is planned to be completed in 6 month, between 1 December 2024 and 31 May 2025.

### Eligibility criteria

#### Participants

Studies that investigated mainstreaming or integration of programmes and/or interventions against NTDs into the national health systems both from the perspectives of the implementer and endemic communities will be included. Mainstreaming or integration initiatives against all conditions that are included in the WHO list of NTDs will be of interest (buruli ulcer; chagas disease; dengue and chikungunya; dracunculiasis; echinococcosis; foodborne trematodiases; human African trypanosomiasis; leishmaniasis; leprosy; lymphatic filariasis; mycetoma, chromoblastomycosis and other deep mycoses; noma; onchocerciasis; rabies; scabies and other ectoparasitoses; schistosomiasis; soil-transmitted helminthiases; snakebite envenoming; taeniasis/cysticercosis; trachoma; and yaws).

#### Concept

The concept that we plan to explore in this scoping review is the mainstreaming of programmes and/or interventions against NTDs into the national health systems. WHO recommended five core strategic interventions to accelerate the prevention, control, elimination and eradication of NTDs: (1) innovative and intensified disease management, (2) preventive chemotherapy, (3) vector control, (4) veterinary public health and (5) provision of safe water, sanitation and hygiene.^1^ Morbidity management and disability prevention will be considered as part of the intensified disease management strategy. Integration or mainstreaming of diagnostics or screening, monitoring, evaluation and reporting activities will also be included in this review. Studies that explore partial or full mainstreaming or integration of any interventions against NTDs that occur at any level and building block of the health system will be included. For instance, studies on financial mainstreaming at the national level as well as integration of any diagnostic or screening activities against NTD at the primary healthcare or community level will be included. However, literature that refer to the integration, grouping or packaging of NTD interventions or any other healthcare activities outside of the national health system will be excluded. Our aim for this scoping review is to unpack implementation practices that would contribute to achieving UHC by strengthening the capacity of national health systems in delivering interventions through existing infrastructures, and thereby improve the sustainability and efficiency of interventions, and create equitable access of patients to all aspects of treatment, care and support against NTDs.^1^

#### Context

In this scoping review, we will include evidence that originates from any NTD endemic setting. We, however, recognise the differences in health system organisation across settings which will influence the interpretation and generalisability of results. We will take caution in interpreting the evidence that originates from such diverse settings. We will include studies published from the year 1995 to the present date as major events that can provide invaluable learning on NTD control have occurred at or after that time. In 1995, the African Programme for Onchocerciasis Control, a global partnership which brought together endemic African countries, donors, non-governmental development organisations (NGDOs), the private sector and affected communities, was launched, with the objective of controlling onchocerciasis in the remaining endemic countries in Africa using self-sustaining community-directed treatment with ivermectin with donations from Merck.^7^ In 1996, WHO launched the WHO Alliance for the Global Elimination of Trachoma by the year 2020.^8^ In 2000, the multidrug therapy for leprosy with donations from Novartis, and the Global Programme to Eliminate Lymphatic Filariasis were launched.^9^

#### Types of sources

This scoping review will primarily consider research articles of any methodology, series and reports, published in any peer-reviewed platforms. In addition, WHO, government, government implementing partners reports and university student thesis and dissertations published in their websites will be included. The research articles will include both experimental and quasi-experimental study designs including randomised controlled trials, non-randomised controlled trials, before and after studies and interrupted time-series studies, analytical observational studies including prospective and retrospective cohort studies, case-control studies and analytical cross-sectional studies, descriptive observational study designs including case series, individual case reports, descriptive cross-sectional studies, pilot studies and programme evaluations. Qualitative studies will also be considered that focus on qualitative data including, but not limited to, designs such as phenomenology, grounded theory, ethnography, qualitative description and action research. Publications that will be excluded from this scoping review will include those without full text, conference abstracts, expert opinion/editorial/letter/commentaries, study protocols, policies, guidelines, manuals, blogs or any other material from non-peer-reviewed platforms (except those published in WHO, government, non-government implementing partners and university websites). Systematic reviews will also not be included. However, we will screen the reference lists of systematic reviews for relevant studies.

### Search strategy

The search strategy will aim to identify published studies and WHO, government, government implementing partners and university websites and will be conducted by an information specialist (IG). The text words contained in the titles and abstracts of relevant articles, and the index terms used to describe the articles were used to develop the search strategies for this review. The following databases were searched from 1995 to 30 October 2024 (online supplemental Annex 1): MEDLINE (Ovid), Embase (Ovid), Global Health (Ovid), Cochrane Database of Systematic Reviews and CENTRAL on the Cochrane Library (Wiley) and Global Index Medicus.

The searches were limited to reports in English language only. There will not be an exclusion on publication status; therefore, all documents that meet the inclusion criteria in their published, in press, in progress and preprint state will be included.

We will also check for reference lists and bibliographies of the identified articles to search for citations not identified by the database search. Non-indexed literature of relevance will be searched from WHO, government, government NTD programme implementing partner and universities websites. The list of the NTD programme implementing partners worldwide will be sought from WHO.

### Source of evidence selection

Following the search, all identified citations will be collated and uploaded into EndNote V.20.4. Duplicates will be removed. Records will then be imported into Covidence (https://www.covidence.org/), where any remaining duplicates will be identified and removed. Titles and abstracts will be screened by two or more independent reviewers for assessment against the inclusion criteria for the review. Potentially relevant sources will be retrieved in full, and their citation details imported. A record without an abstract will be included for full-text retrieval.

The full text of selected citations will be assessed in detail against the inclusion criteria by two or more independent reviewers. Reasons for the exclusion of sources of evidence in full text that do not meet the inclusion criteria will be recorded and reported in the appendices of the final scoping review report. Any disagreements that arise between the reviewers at each stage of the selection process will be resolved through discussion, or with an additional reviewer/s. If the two reviewers’ discussion does not resolve the disagreement, the third reviewer’s decision will remain valid. The results of the search and the study inclusion process will be reported in full in the final scoping review and presented in a PRISMA flow diagram.^10^

Both the screening and selection process will be piloted on 5% (or a minimum of five records) of the records imported between all reviewers. Results of the piloting will then be compared; more piloting would occur if the agreement reached is less than 70% between all reviewers.^11^ Following the screening piloting, each of the remaining records will be screened independently by at least two people. Similarly following the selection piloting, each of the remaining records will be screened in detail against the inclusion criteria by at least two independent reviewers. The reason for exclusion at selection may be due to the abstract without any full text, study protocol, expert opinion/editorial/letter, not NTD related, and main agenda is not mainstreaming or integration into the national health system.

### Data extraction & charting

Data will be extracted from papers that meet the eligibility criteria following the selection process at the full-text level by two or more independent reviewers using a custom data charting form in Covidence (https://www.covidence.org/). The data items to be extracted are summarised in table 1. The data extraction form with the data items is provided in online supplemental Annex 2. The data extraction process will be piloted by three reviewers on two reports. The form will be modified and revised as necessary during the process of extracting data from each included evidence source. Any modifications will be detailed in the final scoping review report. If appropriate, authors of papers will be contacted to request missing or additional data, where required. Three attempts of contact will be made via email. The quality of evidence will be assessed using the JBI suite of critical appraisal tools (https://jbi.global/critical-appraisal-tools).

**Table 1:** Data items to be extracted during the data charting process

Section	Data item category	Specific data items and instructions
Admin	Date of data collection	
	Data collector name	
	Study citation	
General study/publication characteristics	Study ID	
	Publication year	
	Publication type	Journal article, magazine article, preprint, reports, thesis, other
	Lead authors contact details	Email address where available, otherwise postal address.
Research aims and concepts	Aim	Provide a brief sentence describing the aim of the publication
	Study concepts	Provide the name of the concept studied. Eg, integration, mainstreaming, transitioning.
Methods	Participant characteristics	Sample size, mean age, age range, sex, setting, socioeconomic characteristics, sex of participants, eligibility criteria
	Research methods	Quantitative, qualitative, mixed
	Data type	Primary data, secondary data
	Research design/approach	Intervention study (RCT/NRSI), observational study (cohort/cross-sectional/case-control), both,
	Study type	RCT (randomised controlled trial) NRSI (non-randomised study of interventions) Cohort study Cross-sectional study Case-control study Other study type
	Condition/disease of interest (NTDs)	Report the number of the conditions/diseasesReport n per condition
Results	Mainstreaming/integration intervention characteristics	Name or description of the integrated/mainstreamed activity/intervention NTD intervention category Dimension of integration or mainstreaming (as per the Project INTEGRATE framework) The health system framework (PHCPI* framework) that the mainstreaming or integration occurred Level of health system that the mainstreaming or integration occurred Targeted population for the intervention
	Intervention effect/impact	Description or measure of the effectiveness of the mainstreaming/integration for each intervention Intensity of integration/mainstreaming Promising practices or facilitators for each intervention Challenges or barriers for each intervention Benefits for each intervention Risks or side effects for each intervention
	Equity	Description of any difference in mainstreaming/integration outcome across different groups of the population Factors that facilitated or prevented in achieving equity in mainstreamed/integrated care for each intervention
Research gaps and challenges	Reported research gaps/limitations	Provide a short descriptive summary of the publication’s reported research gap or limitations
Recommendations	Reported recommendations	Provide a short descriptive summary of the publication’s reported recommendations
Key findings and conclusions	Reported key findings	Provide a summary of the publication’s reported key findings
	Conclusions	Provide a summary of the publication’s reported conclusions

* PHCPI: Primary Health Care Performance Initiative, https://www.improvingphc.org/phcpi-conceptual-framework.

### Data analysis and presentation

Description of the publications included in this scoping review will be supported with a flow diagram and tables. The data presentation will contain three broad sections: In the first section, we will describe the results of the search strategy and selection process, including a PRISMA flow diagram; in the second section, we will present key information or evidence relevant to mainstreaming of interventions against NTDs into the health system, guided by the data items indicated in table 1^5^; lastly, we will identify and summarise the gaps in the literature on mainstreaming and integration control interventions for NTDs.

We will conduct a thematic analysis as follows. We will summarise and describe the different approaches to mainstreaming according to the Project INTEGRATE framework.^12^ Contextual differences such as health system organisation across settings will be noted to guide the interpretation of the evidence presented. A summary of how the effectiveness of mainstreaming/integration was assessed and key lessons in the form of challenges and promising practices that will limit or facilitate mainstreaming of interventions against NTDs will be presented in a table guided by the Primary Health Care Performance Initiative conceptual framework (system, inputs, service delivery, outputs and outcomes).^13^ When applicable, we will also report the extent to which equity is considered, and key facilitators and barriers to achieving health equity in implementing mainstreaming or integration. Gaps in the literature regarding mainstreaming or integration of programmes or interventions against NTDs will be presented by comparison with the current disease burden of NTDs and considering the quality and depth of the data available on promising practices, challenges and successes.

### Patient and public involvement

None.

### Ethics and dissemination

Ethical approval will not be sought as our review will only include published data. Data will be handled and reported with transparency and integrity to reduce bias and ensure accuracy, consistency, reliability and comprehensiveness.

The review will be conducted as part of a four-phase exploratory implementation research to generate evidence on context-specific strategies, operational capacity and readiness of the Ethiopia primary healthcare system to mainstream and sustainably transition NTD campaign interventions, funded by the Bill and Melinda Gates Foundation to the Task Force for Global Health’s Health Campaign Effectiveness Program. The findings will be published in a peer-reviewed journal and will be disseminated at national and international research and programme review platforms. The research team is well placed to do this as it involves leaders of NTD elimination programmes within Ethiopia and globally.

## supplementary material

10.1136/bmjopen-2024-090252 online supplemental file 1

10.1136/bmjopen-2024-090252 online supplemental file 2
