# Supplementary material for: Mainstreaming control interventions for neglected tropical diseases into the health system: a scoping review protocol
Source: BMJ Open. 2024 Dec 27;14(12):e090252. doi: 10.1136/bmjopen-2024-090252 (PMC11683995; doi:10.1136/bmjopen-2024-090252)
Supplement: online supplemental file 1 [file bmjopen-14-12-s001.docx]

**Supplemental Annex 1**

**Search strategy: 30 October 2024**

MEDLINE

1. Neglected Diseases/

2. (neglect$ adj disease$).tw.

3. (neglect$ adj2 tropical adj2 disease$).tw.

4. NTD.tw.

5. Trachoma/

6. (trachoma$ or tracoma$ or trichiasis).tw.

7. onchocerciasis/ or onchocerciasis, ocular/

8. Onchocerciasis.tw.

9. (river adj2 blindness).tw.

10. elephantiasis/ or elephantiasis, filarial/ or non-filarial lymphedema/

11. elephantiasis.tw.

12. (lymphatic adj2 filariasis).tw.

13. podoconiosis.tw.

14. exp Schistosomiasis/

15. schistosomiasis.tw.

16. exp Helminthiasis/

17. helminthiasis.tw.

18. exp Leishmaniasis/

19. leishmaniasis.tw.

20. Dracunculiasis/

21. dracunculiasis.tw.

22. (Guinea adj1 worm).tw.

23. exp Leprosy/

24. leprosy.tw.

25. Chagas Disease/

26. exp Dengue/

27. dengue.tw.

28. Chikungunya Fever/

29. chikungunya.tw.

30. Trypanosomiasis, African/ or Trypanosomiasis/

31. trypanosomiasis.tw.

32. Rabies/

33. rabies.tw.

34. Scabies/

35. scabies.tw.

36. (Buruli adj1 ulcer$).tw.

37. Mycetoma/

38. mycetoma.tw.

39. Yaws/

40. yaws.tw.

41. ((chemotherap$ or chemoprevention) adj5 (neglected adj1 tropical adj1 disease$)).tw.

42. Echinococcosis/

43. echinococcosis.tw.

44. Trematode Infections/

45. trematodiases.tw.

46. Chromoblastomycosis/

47. Chromoblastomycosis.tw.

48. Noma/

49. noma.tw.

50. Snake Bites/

51. (snake adj2 (bite or envenom$)).tw.

52. Taeniasis/

53. taeniasis.tw.

54. Cysticercosis/

55. cysticercosis.tw.

56. or/1-55

57. (mass adj1 drug adj1 administrat$).tw.

58. 56 and 57

59. 56 or 58

60. (integrat$ adj10 control adj1 program$).tw.

61. (integrat$ adj5 health$ adj3 system$).tw.

62. (integrat$ adj5 vertical).tw.

63. (mainstreaming or transitioning or assimilation or embedding or routinisation or merging).tw.

64. (morbidity adj3 management adj3 disability adj3 prevention).tw.

65. MMDP.tw.

66. or/60-65

67. 59 and 66

Embase

1. Tropical Disease/

2. Neglected Disease/

3. (neglect$ adj disease$).tw.

4. (neglect$ adj2 tropical adj2 disease$).tw.

5. NTD.tw.

6. Trachoma/

7. (trachoma$ or tracoma$ or trichiasis).tw.

8. Onchocerciasis/ or Ocular onchocerciasis/

9. Onchocerciasis.tw.

10. Elephantiasis/ or Lymphatic filariasis/ or Podoconiosis/ or Wuchereria bancrofti/

11. elephantiasis.tw.

12. (lymphatic adj2 filariasis).tw.

13. podoconiosis.tw.

14. exp Schistosomiasis/

15. schistosomiasis.tw.

16. exp Helminthiasis/

17. helminthiasis.tw.

18. exp Leishmaniasis/

19. leishmaniasis.tw.

20. Dracunculiasis/

21. dracunculiasis.tw.

22. (Guinea adj1 worm).tw.

23. Leprosy/

24. leprosy.tw.

25. exp Chagas disease/

26. Dengue/

27. dengue.tw.

28. Chikungunya/

29. chikungunya.tw.

30. African trypanosomiasis/ or Trypanosomiasis/ or Experimental trypanosomiasis/

31. trypanosomiasis.tw.

32. Rabies/

33. rabies.tw.

34. Scabies/

35. scabies.tw.

36. Buruli ulcer/

37. (Buruli adj1 ulcer$).tw.

38. Mycetoma/

39. mycetoma.tw.

40. Yaws/

41. yaws.tw.

42. ((chemotherap$ or chemoprevention) adj5 (neglected adj1 tropical adj1 disease$)).tw.

43. or/1-42

44. (mass adj1 drug adj1 administrat$).tw.

45. 43 and 44

46. 43 or 45

47. (integrat$ adj10 control adj1 program$).tw.

48. (integrat$ adj5 health$ adj3 system$).tw.

49. (integrat$ adj5 vertical).tw.

50. (mainstreaming or transitioning or assimilation or embedding or routinisation or merging).tw.

51. (morbidity adj3 management adj3 disability adj3 prevention).tw.

52. MMDP.tw.

53. or/47-52

54. 46 and 53

Global Health

1. tropical diseases/ or neglected tropical diseases/

2. (neglect$ adj disease$).tw.

3. (neglect$ adj2 tropical adj2 disease$).tw.

4. NTD.tw.

5. Trachoma/

6. (trachoma$ or tracoma$ or trichiasis).tw.

7. onchocerciasis/

8. Onchocerciasis.tw.

9. elephantiasis/ or filariasis/ or lymphatic filariasis/

10. elephantiasis.tw.

11. (lymphatic adj2 filariasis).tw.

12. vector-borne diseases/

13. podoconiosis.tw.

14. exp Schistosomiasis/

15. schistosomiasis.tw.

16. helminthoses/ or helminths/

17. helminthiasis.tw.

18. exp Leishmaniasis/

19. leishmaniasis.tw.

20. Dracunculiasis/

21. dracunculiasis.tw.

22. (Guinea adj1 worm).tw.

23. exp Leprosy/

24. leprosy.tw.

25. Chagas' disease/

26. exp Dengue/

27. dengue.tw.

28. chikungunya virus/

29. chikungunya.tw.

30. trypanosomiasis/ or African trypanosomiasis/

31. trypanosomiasis.tw.

32. Rabies/

33. rabies.tw.

34. Scabies/

35. scabies.tw.

36. Buruli ulcer/

37. (Buruli adj1 ulcer$).tw.

38. Mycetoma/

39. mycetoma.tw.

40. Yaws/

41. yaws.tw.

42. ((chemotherap$ or chemoprevention) adj5 (neglected adj1 tropical adj1 disease$)).tw.

43. or/1-42

44. (mass adj1 drug adj1 administrat$).tw.

45. 43 and 44

46. 43 or 45

47. (integrat$ adj10 control adj1 program$).tw.

48. (integrat$ adj5 health$ adj3 system$).tw.

49. (integrat$ adj5 vertical).tw.

50. (mainstreaming or transitioning or assimilation or embedding or routinisation or merging).tw.

51. (morbidity adj3 management adj3 disability adj3 prevention).tw.

52. MMDP.tw.

53. or/47-52

54. 46 and 53

Cochrane Database of Systematic Reviews and CENTRAL on the Cochrane Library

#1 MeSH descriptor: [Neglected Diseases] this term only

#2 neglect* NEXT disease*

#3 neglect* NEAR/2 tropical NEAR/2 disease*

#4 NTD

#5 MeSH descriptor: [Trachoma] this term only

#6 trachoma* or tracoma* or trichiasis

#7 MeSH descriptor: [Onchocerciasis] this term only

#8 MeSH descriptor: [Onchocerciasis, Ocular] this term only

#9 Onchocerciasis

#10 MeSH descriptor: [Elephantiasis] this term only

#11 MeSH descriptor: [Elephantiasis, Filarial] this term only

#12 MeSH descriptor: [Non-Filarial Lymphedema] this term only

#13 elephantiasis

#14 lymphatic NEAR/2 filariasis

#15 podoconiosis

#16 MeSH descriptor: [Schistosomiasis] this term only

#17 schistosomiasis

#18 MeSH descriptor: [Helminthiasis] explode all trees

#19 helminthiasis

#20 MeSH descriptor: [Leishmaniasis] explode all trees

#21 leishmaniasis

#22 MeSH descriptor: [Dracunculiasis] this term only

#23 dracunculiasis

#24 Guinea NEXT worm

#25 MeSH descriptor: [Leprosy] explode all trees

#26 leprosy

#27 MeSH descriptor: [Chagas Disease] this term only

#28 MeSH descriptor: [Dengue] explode all trees

#29 dengue

#30 MeSH descriptor: [Chikungunya Fever] this term only

#31 chikungunya

#32 MeSH descriptor: [Trypanosomiasis, African] this term only

#33 MeSH descriptor: [Trypanosomiasis] this term only

#34 trypanosomiasis

#35 MeSH descriptor: [Rabies] this term only

#36 rabies

#37 MeSH descriptor: [Scabies] this term only

#38 scabies

#39 Buruli NEXT ulcer*

#40 MeSH descriptor: [Mycetoma] this term only

#41 mycetoma

#42 MeSH descriptor: [Yaws] this term only

#43 yaws

#44 (chemotherap* or chemoprevention) NEAR/5 (neglected NEXT tropical NEXT disease*)

#45 #1 OR #2 OR #3 OR #4 OR #5 OR #6 OR #7 OR #8 OR #9 OR #10 OR #11 OR #12 OR #13 OR #14 OR #15 OR #16 OR #17 OR #18 OR #19 OR #20 OR #21 OR #22 OR #23 OR #24 OR #25 OR #26 OR #27 OR #28 OR #29 OR #30 OR #31 OR #32 OR #33 OR #34 OR #35 OR #36 OR #37 OR #38 OR #39 OR #40 OR #41 OR #42 OR #43 OR #44

#46 mass NEXT drug NEXT administrat*

#47 #45 AND #46

#48 #45 or #47

#49 integrat* NEAR/10 control NEXT program*

#50 integrat* NEAR/5 health* NEAR/3 system*

#51 integrat* NEAR/5 vertical

#52 mainstreaming OR transitioning OR assimilation OR embedding OR routinisation OR merging

#53 morbidity NEAR/3 management NEAR/3 disability NEAR/3 prevention

#54 MMDP

#55 #49 OR #50 OR #51 OR #52 OR #53 OR #54

#56 #48 AND #55

Global Index Medicus

("mainstreaming" OR "transitioning" OR "assimilation" OR "embedding" OR "routinisation" OR merging) AND ("neglected tropical disease")
