# Supplementary material for: Mainstreaming control interventions for neglected tropical diseases into the health system: a scoping review protocol
Source: BMJ Open. 2024 Dec 27;14(12):e090252. doi: 10.1136/bmjopen-2024-090252 (PMC11683995; doi:10.1136/bmjopen-2024-090252)
Supplement: online supplemental file 2 [file bmjopen-14-12-s002.docx]

**Supplemental Annex 2: Data extraction form with data items**

| **Section** | **Data item category** | **Specific data items and instructions** | **Data format in Covidence** |
| --- | --- | --- | --- |
| Admin | Date of data collection |  | Collected automatically in Covidence |
|  | Data collector name |  | Collected automatically in Covidence |
|  | Study citation |  | Collected automatically in Covidence |
| General study/publication characteristics | Study ID | Please provide in format Author Year eg Adgo 2023 | Text field |
|  | Publication year |  | Text field |
|  | Publication type | - Journal article - Magazine article - Preprint - Reports - Thesis - Other | Single choice |
|  | Lead authors contact details | Email address where available, otherwise postal address. | Text field |
| Research aims and concepts | Aim | Provide a brief sentence describing the aim of the publication | Text field |
|  | Study concepts | Provide the name of concept studied. E.g. integration, mainstreaming, transitioning, etc. | Checkbox |
| Methods | Participant characteristics | - N (total sample): report the number of participants in the total sample, including excluded participants - N (final sample): report the number of participants included in the final sample - Mean age: write the reported average age of participants with (SD) - Age range of participants - Sex of participants - Setting or country of data collection - Socio-economic characteristics - Eligibility criteria - Other relevant participant characteristics   Note: for age-related entities, please denote with the letters m for months, y for years, d for days, and wks for weeks old | Table |
|  | Research methods | - Quantitative - Qualitative - Mixed | Single choice |
|  | Data type | - Primary data - Secondary data | Checkbox |
|  | Research design/approach | - Intervention study (RCT/NRSI) - Observational study (cohort/cross-sectional/case-control) - Both | Single choice |
|  | Study type | - RCT (randomised controlled trial) - NRSI (non-randomised study of interventions) - Cohort study - Cross-sectional study - Case-control study - Other study type | Checkboxes (select the type(s) of study type |
|  | Condition/disease of interest (NTDs) | Report the number of the conditions/diseases  Report the names of the conditions (Any condition that are included in the WHO list of NTDs. List of all NTDs)   - Name of the first condition - Name of the second condition, etc.   Report n per condition | Table |
| Results | Mainstreaming/integration intervention characteristics | 1. Name or description of the integrated/mainstreamed activity/intervention for each intervention: intervention 1, intervention 2, intervention 3, etc. 2. NTD intervention category. Data will be extracted to categorise the NTD intervention type that the study focused on:  - Innovative and intensified disease management including morbidity management and disability prevention (MMDP) - Preventive chemotherapy - Vector control - Veterinary public health, and - Provision of safe water, sanitation, and hygiene  1. Dimension of integration or mainstreaming (as per the Project INTEGRATE framework). Data will be extracted to categorise the integration or mainstreaming into one or more of the following dimensions:  - Person centred care - Clinical integration - Professional integration - Organisational integration - Systemic integration - Functional integration - Normative integration  1. The health system framework (PHCPI* framework) that the mainstreaming or integration occurred:  - System - Inputs - Service Delivery  1. Level of health system that the mainstreaming or integration occurred:  - Primary - Secondary - Tertiary  1. Targeted population for the intervention:  - Community - Healthcare workers - Health are leaders, etc. | Table |
|  | Intervention effect/impact | 1. Description or measure of effectiveness of the mainstreaming/integration for each intervention 2. Intensity of integration/mainstreaming 3. Promising practices or facilitators for each intervention 4. Challenges or barriers for each intervention 5. Benefits for each intervention 6. Risks or side effects for each intervention | Table, by intervention |
|  | Equity | 1. Description of any difference in mainstreaming/integration outcome across different groups of the population (such as based on gender, age, socio-economic status, geography, etc.) for each intervention 2. Factors that facilitated or prevented in achieving equity in mainstreamed/integrated care for each intervention | Table, by intervention |
| Research Gaps & Challenges | Reported research gaps/limitations | Provide a short descriptive summary of the publication’s reported research gap or limitations | Text field |
| Recommendations | Reported recommendations | Provide a short descriptive summary of the publication’s reported recommendations | Text field |
| Key Findings & Conclusions | Reported key findings | Provide a summary of the publication’s reported key findings | Text field |
|  | Conclusions | Provide a summary of the publication’s reported conclusions | Text field |

* PHCPI: Primary Health Care Performance Initiative, <https://www.improvingphc.org/phcpi-conceptual-framework>
