# Supplementary material for: Mainstreaming control interventions for neglected tropical diseases into the health system: a scoping review protocol
Source: BMJ Open. 2024 Dec 27;14(12):e090252. doi: 10.1136/bmjopen-2024-090252 (PMC11683995; doi:10.1136/bmjopen-2024-090252)
Supplement: Supplementary file 3 [file bmjopen-2024-090252.reviewer_comments.pdf]

PEER REVIEW HISTORY

BMJ Open publishes all reviews undertaken for accepted manuscripts. Reviewers are asked to complete a checklist review form and are provided with free text boxes to elaborate on their assessment. These free text comments are reproduced below.

ARTICLE DETAILS

Title (Provisional)

Mainstreaming control interventions for neglected tropical diseases into the health system: a scoping review protocol

Authors

Bilchut, Awraris Hailu; Gebre, Teshome; Seife Gebretsadik, Fikre; Enbiale, Wendemagegn; Tamiru, Adugna; Yohanes, Tsegaye; Yohannes, Amsayaw; Adane, Yosef; Merdekios, Behailu; Kebede, Biruck; Kebede, Fikreab; Deribe, Kebede; Evans, Jennifer; Burton, Matthew J; Habtamu, Esmael

---

VERSION 1 - REVIEW

**Reviewer**	**1**
**Name**	**Akinsolu, Folahanmi**
**Affiliation**	**Lead City University, Public Health**
**Date**	**04-Aug-2024**
**COI**	**I have no competing interests**

Are the methods described sufficiently to allow the study to be repeated?

The methods section should provide a more detailed description of the specific data items to be extracted during the data charting process. Additionally, the exact search strategy for each database should be included to ensure the reproducibility of the study.

Are the study limitations discussed adequately?

The protocol should address potential limitations such as language restrictions (only including English-language publications) and the exclusion of non-peer-reviewed literature, which may lead to publication bias. Discussing these limitations would provide a more comprehensive view of the study's potential constraints and the robustness of its findings.

Review Comments

General Comments
The study protocol presents a comprehensive plan to conduct a scoping review on mainstreaming NTD interventions into healthcare systems. The research addresses a critical gap in the literature, given the global health focus on integrating NTD control into national health systems. However, several areas need attention to enhance the protocol's clarity and robustness.

Strengths

1. **Timely and Relevant Topic:** The study addresses the integration of NTD interventions into healthcare systems, a critical area for achieving universal health coverage.
2. **Comprehensive Methodology:** The use of JBI methodology and PRISMA-ScR guidelines ensures a systematic and transparent review process.
3. **Experienced Team:** The involvement of experienced evidence synthesis and information specialists strengthens the protocol's credibility.

Areas for Improvement

1. **Introduction Clarity:**
   - The introduction should provide more specific examples of NTD interventions that have been successfully mainstreamed, if available.
   - Clarify how the scoping review will specifically contribute to the objectives outlined in the WHO NTD roadmap.
2. **Methods:**
   - The eligibility criteria should explicitly state the types of study designs to be included or excluded (e.g., randomized controlled trials, observational studies).
   - The data extraction process mentions a "custom data charting form" but does not provide details on the specific data items to be extracted. Including a sample of the form or more detailed description would be beneficial.
3. **Search Strategy:**
   - The search strategy for each database should be provided in more detail, including specific keywords and index terms. This will ensure the search is reproducible.
   - Consider including grey literature sources beyond WHO and government websites to capture a broader range of relevant studies.
4. **Data Analysis:**
   - The protocol should provide more details on how the data will be synthesized. For example, will a thematic analysis be conducted? How will the data be categorized and summarized?
   - Explain how the quality of the included studies will be assessed, even if a formal risk of bias assessment is not planned.
5. **Limitations:**
   - Acknowledge potential limitations of the scoping review, such as language restrictions and the exclusion of non-peer-reviewed literature, which may lead to publication bias.
6. **Ethics and Dissemination:**
   - While ethical approval is not required, the protocol should outline any ethical considerations related to the handling and reporting of data.

Detailed Comments

- **Page 2, Line 1-5:** The rationale for focusing on the integration of NTD interventions should be more explicitly linked to specific health outcomes or system efficiencies expected from such integration.
- **Page 4, Line 15-20:** The eligibility criteria should include a justification for the date range selected (1995 to present).
- **Page 6, Line 25-30:** The process for resolving disagreements between reviewers should be detailed, including the role of the additional reviewer.
- **Page 8, Line 10-15:** The data charting form should include an example of how data will be extracted and categorized.

Conclusion

The study protocol outlines a well-structured approach to conducting a scoping review on mainstreaming NTD interventions into healthcare systems. By addressing the areas for improvement, the protocol can be strengthened to ensure a comprehensive and robust review process. The recommendations provided aim to enhance the clarity, reproducibility, and rigor of the scoping review, aligning with BMJ Open's standards for study protocols.

**Reviewer**	**2**
**Name**	**Krentel, Alison**
**Affiliation**	**Bruyère Research Institute**
**Date**	**01-Oct-2024**

COI **I have ongoing research in Ethiopia that collaborates with the NTD program at the Ethiopia Ministry of Health.**

This is an interesting topic for a scoping review. The choice of a scoping review is appropriate as the number of publications on the subject is presently unknown.

A few comments for your consideration:

Comment on language – recommend shift to more empowering language – e.g., people living in marginalized situations as opposed to marginalized populations. E.g., line 155 – use of 'beneficiary' shift to 'endemic communities' or 'end-user' or other similar terms.

Verify the number affected – line 92 – have seen recent numbers as 1.6 billion

Paper refers to the 'general health care system' – I wonder why the authors do not use 'the health system' simply?

There are some edits required (e.g., lines 109-112, 165-6, 169, 174, 175-177). There are more sections that would benefit from an additional editorial review as the language needs clarity.

Lines 113, 148 – if the systematic review has been registered – please indicate the reference to the registered protocol.

Was the search strategy developed or reviewed by a librarian? If so, please indicate. If not, then I would suggest that the authors have a librarian review the search strategy and criteria. For example, are evidence from all countries included? In the search strategy, it doesn't look like there are any search terms related to country. Also, in reviewing the search strategy, I am not sure you will capture programs where mainstreaming has occurred but they haven't used this term, or integration. Has there been an exhaustive review of the possible terms for this?

Line 175-77: Why exclude grouping or packaging of diseases? This seems to be an appropriate and important response to integration of NTDs within the health system. The review will miss valuable evidence as to whether this approach may be an effective approach to integration. It is an assumption by the authors that this combination is only used outside of the health system.

Intervention characteristics – where does morbidity management and disability prevention fit within these characteristics? This is important as it provides an excellent opportunity for health system integration. If MMDP fits within IDM for the purposes of this review, it will be important to state that in the paper.

VERSION 1 - AUTHOR RESPONSE

Reviewer: 1

General Comments

The study protocol presents a comprehensive plan to conduct a scoping review on mainstreaming NTD interventions into healthcare systems. The research addresses a critical gap in the literature, given the global health focus on integrating NTD control into national health systems. However, several areas need attention to enhance the protocol's clarity and robustness.

We are grateful for these insightful comments which have helped us to improve our manuscript. We have provided below a point-by-point account on how we addressed them.

Are the methods described sufficiently to allow the study to be repeated?
The methods section should provide a more detailed description of the specific data items to be extracted during the data charting process. Additionally, the exact search strategy for each database should be included to ensure the reproducibility of the study.

We completely agree with the reviewer on both suggestions.

We have amended Table 1 to be more specific on the data items to be extracted under each category of subject of interest. For instance, under "General study/publication characteristics" we indicated the specific data items such as study ID, authors, study title, publication year, publication type …etc will be extracted.

We have now included an updated the search strategy for each database in Supplemental Annex 1.

Are the study limitations discussed adequately?
The protocol should address potential limitations such as language restrictions (only including English-language publications) and the exclusion of non-peer-reviewed literature, which may lead to publication bias. Discussing these limitations would provide a more comprehensive view of the study's potential constraints and the robustness of its findings.

Please kindly note that we have already indicated the two limitations raised by the esteemed reviewer in the "Strength and Limitation" section on page 3. We could not expand on those sentences at it's the journals requirement to include "…up to five short bullet points, no longer than one sentence each". We have revised the two bullet points to indicate the bias associated with language restrictions and exclusion of non-peer-reviewed literature. We have also included one more potential limitation in relation to the small number of publications that identified that report mainstreaming of NTD interventions. The revised "Strength and Limitation" section on page 3 reads as follows:

- *The search will be guided and implemented by two experienced evidence synthesis and information specialists.*
- *The reviewers will bring diverse perspectives from various population backgrounds and academic disciplines, which helps mitigate potential biases in interpreting the studies under review.*
- *The scoping review will only include those that are published in English language, introducing an element of inclusion bias.*
- *We will only focus on publications and reports on peer reviewed platforms and websites of WHO, universities, government and implementing partners, excluding information produced outside of the traditional publishing and distribution channels, which may lead to publication bias.*
- *A potential limitation could be a small number of publications identified that report mainstreaming of NTD program or interventions.*

1. Introduction Clarity

The introduction should provide more specific examples of NTD interventions that have been successfully mainstreamed, if available.

Although we agree that this would have been a nice addition, we refrain from adding specific examples of NTD interventions that have been successfully mainstreamed as part of this protocol as this is the whole purpose of this scoping review. Such mainstreaming examples will be identified and discussed in detail when we report the results of the scoping review in the near future.

Clarify how the scoping review will specifically contribute to the objectives outlined in the WHO NTD roadmap.

The point that the esteemed reviewer raised is woven in the introduction section. We have now updated the last paragraph of the introduction as follows to address this suggestion.

"The potential evidence to be generated from this scoping review will support NTD endemic countries' efforts to achieve the envisaged targets of the WHO NTD roadmap. It will identify promising practices, challenges, and success from different settings allowing endemic countries to learn from each other on how to intensify cross-cutting actions including the integration, mainstreaming and coordination of interventions, programmes and stakeholders against NTDs".

2. Methods

The eligibility criteria should explicitly state the types of study designs to be included or excluded (e.g., randomized controlled trials, observational studies).

The type of articles to be included and their designs are already indicated in the first submission under the "Types of Sources" section. Please kindly see page 7, lines 336 – 364 in the revised manuscript, tracked changes version.

The data extraction process mentions a "custom data charting form" but does not provide details on the specific data items to be extracted. Including a sample of the form or more detailed description would be beneficial.

The specific data items to be extracted are indicated in Table 1. Our plan is to use a custom chart in Covidence to extract the data. There is no easy way to share a Covidence chart. We, added supplemental annex 2 to showcase the format of the data extraction chart in Covidence with the data items. Please kindly note that this data extraction form is a draft and will be piloted and amended as necessary. Please see page 9, lines 465 – 468.

3. Search Strategy

The search strategy for each database should be provided in more detail, including specific keywords and index terms. This will ensure the search is reproducible.

We have now included the search strategy for all relevant databases in the online supplemental annex 1.

Consider including grey literature sources beyond WHO and government websites to capture a broader range of relevant studies.

The reason why we didn't want to add the grey literature other than WHO, government, and NGO reports is due to the potential familiarity bias introduced by the investigators access to that particular literature in relation to their scope of work and location. It is an avoidable that some grey literature from some settings and conditions will be excluded. That being said, we have now revised the inclusion criteria to include student reports and thesis from university websites (see page 7, line 338). We believe the current eligibility criteria will cover most of the source of the relevant evidence. But we still indicated that the exclusion of other grey literature sources is the limitation of the study.

4. Data Analysis

The protocol should provide more details on how the data will be synthesized. For example, will a thematic analysis be conducted? How will the data be categorized and summarized?

The analysis section now has been updated to clarify how thematic analysis will be conducted, page 10, lines 513 – 525.

Explain how the quality of the included studies will be assessed, even if a formal risk of bias assessment is not planned.

We did not plan to include risk of bias assessment as this is not a requirement for scoping reviews, however, we accept that some assessment of quality would be helpful in our overall assessment of the evidence. We have amended the protocol to include the Joanna Briggs Institute suite of critical appraisal tools as these cover a wide range of study designs.

The following statements are inserted is the "Data Extraction and Charting" section, on page 9, lines 469 – 471 to indicate our quality assessment plan.

"The quality of evidence will be assessed using the Joanna Briggs Institute (JBI) suite of critical appraisal tools (https://jbi.global/critical-appraisal-tools).

5. Limitations

Acknowledge potential limitations of the scoping review, such as language restrictions and the exclusion of non-peer-reviewed literature, which may lead to publication bias.

As indicated above, we have revised the limitation session acknowledging the inclusion and publication bias related with language restriction and the exclusion of non-peer-reviewed literature. Please kindly refer page 3.

6. Ethics and Dissemination

While ethical approval is not required, the protocol should outline any ethical considerations related to the handling and reporting of data.

The following sentence has been included in the Ethical consideration section, page 10, lines 533 – 535.

"Data will be handled and reported with transparency and integrity to reduce bias and ensure accuracy, consistency, reliability and comprehensiveness".

Detailed Comments

- **Page 2, Line 1-5: The rationale for focusing on the integration of NTD interventions should be more explicitly linked to specific health outcomes or system efficiencies expected from such integration.**

In addition to the detailed description in the last paragraph of the introduction on how the evidence on mainstreaming or integration will help countries to achieve the 2030 WHO NTD road map target, we have now added the following sentence on Page 4, lines 144 – 146.

"Integrated and mainstreamed approaches improve sustainability, efficiency, and patients' equitable access to all aspects of treatment, care and support".

- **Page 4, Line 15-20: The eligibility criteria should include a justification for the date range selected (1995 to present).**

The justification for the year range is already indicated in the "Context" section, page 7, lines 322 – 332.

Page 6, Line 25-30: The process for resolving disagreements between reviewers should be detailed, including the role of the additional reviewer.

The process for resolving disagreements is indicated under the "Source of Evidence Selection" section on page 9, lines 443 – 446 as follows:

“Any disagreements that arise between the reviewers in the data charting process will be resolved through discussion, or with an additional reviewer/s. If the two reviewers' discussion does not resolve the disagreement, the third reviewer's decision will remain valid.”.

- **Page 8, Line 10-15: The data charting form should include an example of how data will be extracted and categorized.**

As indicated in earlier responses, data will be extracted in Covidence. Tey data items to be extracted are shown in Table 1, and the format of the custom data extraction chart to be developed in Covidence is provided as supplemental annex 2. This will be piloted and amended as appropriate. See lines 465 – 468, on page 9.

Conclusion
The study protocol outlines a well-structured approach to conducting a scoping review on mainstreaming NTD interventions into healthcare systems. By addressing the areas for improvement, the protocol can be strengthened to ensure a comprehensive and robust review process. The recommendations provided aim to enhance the clarity, reproducibility, and rigor of the scoping review, aligning with BMJ Open's standards for study protocols.

Thank you.

Reviewer: 2
Comments to the Author:

This is an interesting topic for a scoping review. The choice of a scoping review is appropriate as the number of publications on the subject is presently unknown.

Thank you.

A few comments for your consideration:
Comment on language – recommend shift to more empowering language – e.g., people living in marginalized situations as opposed to marginalized populations. E.g., line 155 – use of ‘beneficiary’ shift to ‘endemic communities’ or ‘end-user’ or other similar terms.

We have made the suggested and other edits across the manuscript.

Verify the number affected – line 92 – have seen recent numbers as 1.6 billion.

The most recent WHO report (2024) indeed indicate that number of people requiring interventions are 1.62 billion. This has now been updated in the manuscript.

Paper refers to the 'general health care system' – I wonder why the authors do not use 'the health system' simply?

Both refer largely to the same thing. But "the health system", unlike the "general health care system" may include the private health care system in some contexts. We made edits across the manuscript including in the title to change it to the "health system" and at times to the "national health system".

There are some edits required (e.g., lines 109-112, 165-6, 169, 174, 175-177). There are more sections that would benefit from an additional editorial review as the language needs clarity.

We have gone through the whole manuscript to address these and other similar sentences that needs editing and clarity.

Lines 113, 148 – if the systematic review has been registered – please indicate the reference to the registered protocol.

The following reference number is indicated on page 4, line 161:

PROSPERO, registration number: CRD42022362624.

Was the search strategy developed or reviewed by a librarian? If so, please indicate. If not, then I would suggest that the authors have a librarian review the search strategy and criteria. For example, are evidence from all countries included? In the search strategy, it doesn't look like there are any search terms related to country. Also, in reviewing the search strategy, I am not sure you will capture programs where mainstreaming has occurred but they haven't used this term, or integration. Has there been an exhaustive review of the possible terms for this?

The search strategy has been developed by an experienced information specialist at LSHTM, UK. The review will be assessing NTDs in any country of interest, therefore the search has not included any details on limiting to particular countries. As we will assess studies on NTDs in any relevant country we did not find it necessary to list all the potential country names in the search.

We have done an exhaustive list of terms used. Other terms rarely used to indicate mainstreaming are "transitioning", "assimilation", "embedding", "routinisation", and "merging". We included these terms in the updated search strategy submitted as online supplemental annex 1.

Line 175-77: Why exclude grouping or packaging of diseases? This seems to be an appropriate and important response to integration of NTDs within the health system. The review will miss valuable evidence as to whether this approach may be an effective approach to integration. It is an assumption by the authors that this combination is only used outside of the health system.

We agree that provision of different services through packaging or grouping is one of the appropriate and important response to integration of NTDs into the health system. We have now revised the eligibility criteria to indicate that integration attempts that occur within the health system will be included; and those that occur outside of the health system will be excluded. Page 6, lines 275 – 281 read as follows to reflect this change.

"Studies that explore partial or full mainstreaming or integration of any interventions against NTDs that occur at any level and building block of the health system will be included. For instance, studies on financial mainstreaming at the national level as well as integration of any diagnostic or screening activities against NTD at the primary health care or community level will be included. However, literature that refer to integration, grouping or packaging of NTD interventions or any other health care activities outside of the national health system will be excluded."

Intervention characteristics – where does morbidity management and disability prevention fit within these characteristics? This is important as it provides an excellent opportunity for health system integration. If MMDP fits within IDM for the purposes of this review, it will be important to state that in the paper.

MMDP is part of the intensified disease management strategy, therefore it will be one of the key concepts that we plan to look at in this review. To make this explicit we have included MMDP:

1) On page 6, line 272 – 273
2) In the search strategy (supplemental annex 1)
3) Data extraction form (supplemental annex 2)
